# 2-Amino-7,7-dimethyl-5-oxo-4-[3-(trifluoro­meth­yl)phen­yl]-5,6,7,8-tetra­hydro-4*H*-chromene-3-carbonitrile

**DOI:** 10.1107/S1600536813004522

**Published:** 2013-02-20

**Authors:** Rajni Kant, Vivek K. Gupta, Kamini Kapoor, D. R. Patil, D. R. Chandam, Madhukar B. Deshmukh

**Affiliations:** a X-ray Crystallography Laboratory, Post-Graduate Department of Physics & Electronics, University of Jammu, Jammu Tawi 180 006, India; b Department of Chemistry, Shivaji University, Kolhapur 416 004 (MS), India

## Abstract

In the title mol­ecule, C_19_H_17_F_3_N_2_O_2_, the fused cyclo­hexene and pyran rings adopt sofa and flattened boat conformations, respectively. The four essentially planar atoms of the pyran ring [maximum deviation = 0.008 (2) Å] form a dihedral angle of 88.13 (9)° with the benzene ring. The F atoms of the trifluoro­methyl group were refined as disordered over three sets of sites in a 0.507 (7):0.330 (7):0.163 (3) ratio. In the crystal, mol­ecules are connected into inversion dimers *via* pairs of N—H⋯N hydrogen bonds and these dimers are further linked by N—H⋯O hydrogen bonds into a two-dimensional network parallel to (100).

## Related literature

For the biological activity of 4*H*-pyran derivatives, see: Bhattacharyya *et al.*(2012 ▶); Khaksar *et al.* (2012 ▶); Fotouhi *et al.* (2007 ▶). For related structures, see: Wang (2011 ▶); Anthal *et al.* (2012 ▶); Kant *et al.* (2013 ▶). For ring conformations, see: Duax & Norton (1975 ▶).
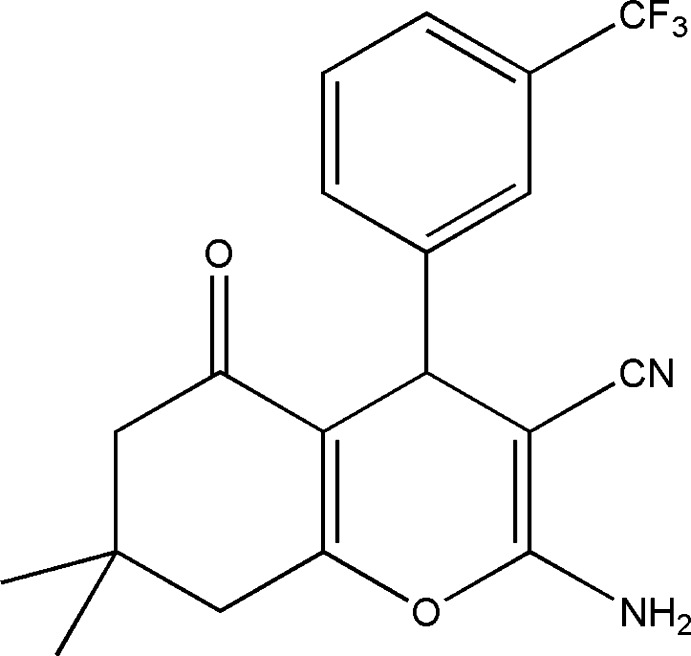


## Experimental

### 

#### Crystal data

C_19_H_17_F_3_N_2_O_2_
*M* *_r_* = 362.35
Monoclinic, 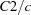

*a* = 23.7543 (6) Å
*b* = 9.3871 (2) Å
*c* = 15.8857 (4) Å
β = 94.704 (2)°
*V* = 3530.33 (15) Å^3^
*Z* = 8
Mo *K*α radiation
μ = 0.11 mm^−1^
*T* = 293 K
0.3 × 0.2 × 0.2 mm

#### Data collection

Oxford Diffraction Xcalibur Sapphire3 diffractometer
Absorption correction: multi-scan (*CrysAlis PRO*; Oxford Diffraction, 2010 ▶) *T*_min_ = 0.766, *T*_max_ = 1.000
40937 measured reflections
3467 independent reflections
2538 reflections with *I* > 2σ(*I*)
*R*_int_ = 0.065

#### Refinement

*R*[*F* ^2^ > 2σ(*F* ^2^)] = 0.061
*wR*(*F* ^2^) = 0.126
*S* = 1.03
3467 reflections
258 parameters
10 restraints
H-atom parameters constrained
Δρ_max_ = 0.26 e Å^−3^
Δρ_min_ = −0.32 e Å^−3^

### 

Data collection: *CrysAlis PRO* (Oxford Diffraction, 2010 ▶); cell refinement: *CrysAlis PRO*; data reduction: *CrysAlis PRO*; program(s) used to solve structure: *SHELXS97* (Sheldrick, 2008 ▶); program(s) used to refine structure: *SHELXL97* (Sheldrick, 2008 ▶); molecular graphics: *ORTEP-3 for Windows* (Farrugia, 2012 ▶) and *PLATON* (Spek, 2009 ▶); software used to prepare material for publication: *PLATON*.

## Supplementary Material

Crystal structure: contains datablock(s) I, global. DOI: 10.1107/S1600536813004522/lh5585sup1.cif

Structure factors: contains datablock(s) I. DOI: 10.1107/S1600536813004522/lh5585Isup2.hkl

Supplementary material file. DOI: 10.1107/S1600536813004522/lh5585Isup3.cml

Additional supplementary materials:  crystallographic information; 3D view; checkCIF report

## Figures and Tables

**Table 1:** Hydrogen-bond geometry (Å, °)

*D*—H⋯*A*	*D*—H	H⋯*A*	*D*⋯*A*	*D*—H⋯*A*
N21—H21*A*⋯N20^i^	0.86	2.17	3.025 (3)	171
N21—H21*B*⋯O2^ii^	0.86	2.10	2.934 (2)	163

Symmetry codes: (i) 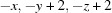; (ii) 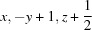.

**Fig. 1.**
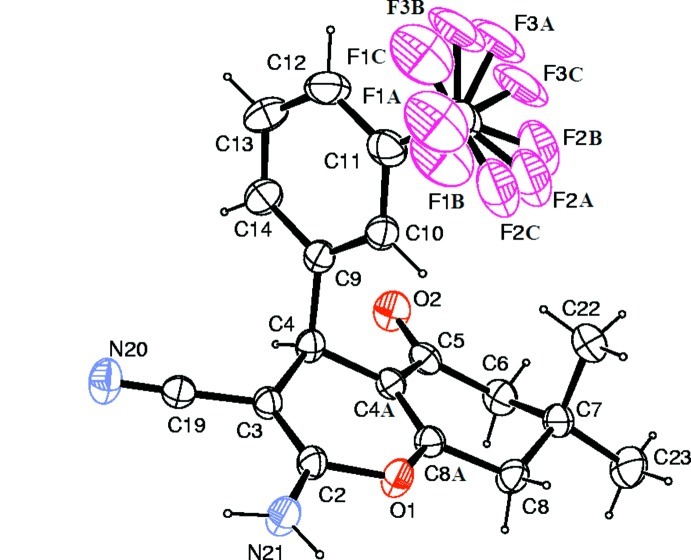
The molecular structure of the title compound with ellipsoids drawn at the 40% probability level. H atoms are shown as small spheres of arbitrary radii. The F atoms are disorded over three sets of sites.

**Fig. 2.**
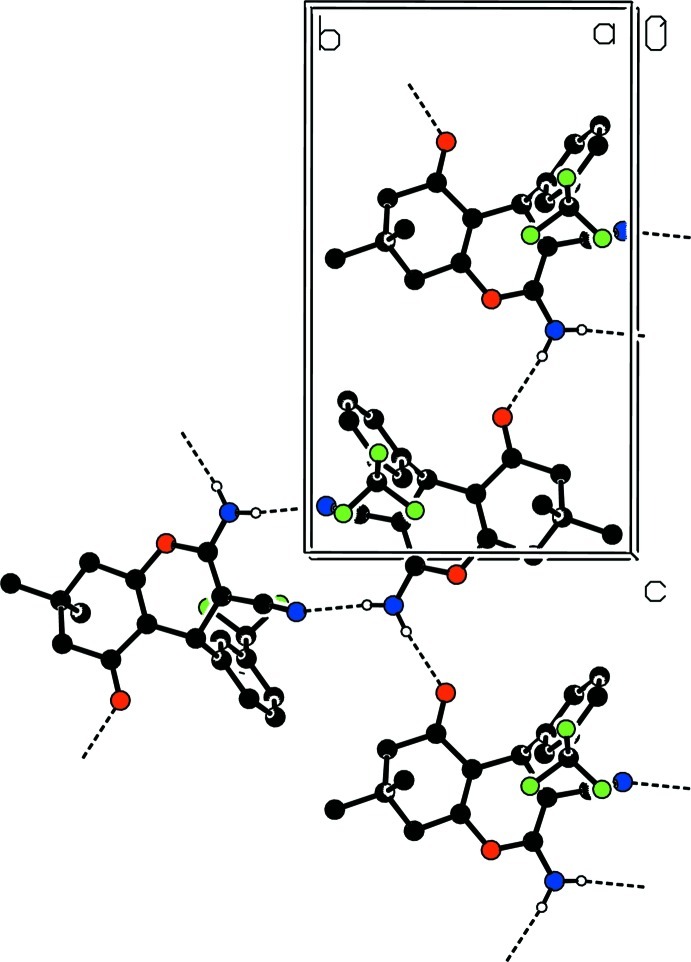
The packing arrangement of molecules viewed along the a axis. The dashed lines show intermolecular N—H···O and N—H···N hydrogen bonds. The disorder is not shown.

## supplementary crystallographic information

### Comment

Polyfunctionalized 4H-pyran derivatives are used as anti-coagulants, anticancer agents, spasmolytics, anti-anaphylactics, anti-microbial and immunomodulating activities (Khaksar *et al.*, 2012; Bhattacharyya *et al.*, 2012). Furthermore, these compounds can be employed as pigments, photoactive materials and used as biodegradable agrochemicals (Fotouhi *et al.*, 2007). In this paper, we report the crystal structure of the title compound, (I).

In (I) (Fig.1), all bond lengths and angles are normal and correspond to those observed in related structures (Wang *et al.*,2011; Anthal *et al.*, 2012; Kant *et al.*,2013). The cyclohexene ring (C5/C6/C7/C8/C8A/C4A) and and pyran ring (O1/C2/C3/C4/C4A/C8A) exhibit sofa and boat conformations, respectively, with asymmetry parameters (ΔCs(C7) = 9.78 & ΔCs(C4) = 2.36, ΔCs(C2-C3)= 9.4)(Duax & Norton, 1975) with atom C7 forming the flap in the cyclohexene ring. The four essentially planar atoms (C2/C3/C4A/C8A) of pyran ring (maximum deviation = -0.008 (2)Å for C8A) form a dihedral angle of 88.13 (9)° with benzene the ring. The F atoms of the trifluoromethyl group were refined as disordered over three sets of sites in a 0.507 (7) : 0.330 (7) : 0.163 (3) ratio. In the crystal, molecules are connected into dimers via N21—H21A···N20^i^ hydrogen bonds and these dimers are further connected by N21—H21B···O2^ii^ (Table 1) hydrogen bonds into a two-dimensional network (Fig. 2) parallel to (100).

### Experimental

In a 50 ml round bottom flask charged with 1mmole of dimedone, 1 mmole of 3-(trifluoromethyl)benzaldehyde and 1 mmole of malononitrile were added. Then 5 ml of aqueous ethanol (1:1) and 20 mol% of NH_4_Cl was added and the reaction mixture stirred for 30-45 min. at 323-328 K. The reaction was monitored by TLC. After completion of the reaction, the mixture was poured onto crushed ice and stirred. The solid precipitated was filtered and recrystallized from ethanol to afford pure product as crystal suitable for X-ray diffraction.

m.p.: 503-504 K, Yield: 82%.

1H NMR (300MHz,DMSO-d6): δ 0.94(s, 3H, CH3), 1.02(s, 3H, CH3), 2.05-2.20(m,2H, CH2), 2.44-2.49(m, 2H, CH2), 4.18(s, 1H, CH), 6.73(s, 2H,NH2), 6.81-6.87(m, 2H, Ar-H), 6.92-6.95(m, 1H, Ar-H), 7.18-7.25(m, 1H, Ar-H).

### Refinement

All H atoms were positioned geometrically and were treated as riding on their parent C/N atoms, with C—H distances of 0.93–0.98 Å, N—H distances of 0.86 Å and with *U*_iso_(H) = 1.2*U*_eq_(C/N) or 1.5*U*_eq_(methyl C).

### Crystal data

**Table:**

C_19_H_17_F_3_N_2_O_2_	*F*(000) = 1504
*M**_r_* = 362.35	*D*_x_ = 1.363 Mg m^−^^3^
Monoclinic, *C*2/*c*	Mo *K*α radiation, λ = 0.71073 Å
Hall symbol: -C 2yc	Cell parameters from 18267 reflections
*a* = 23.7543 (6) Å	θ = 3.4–29.1°
*b* = 9.3871 (2) Å	µ = 0.11 mm^−^^1^
*c* = 15.8857 (4) Å	*T* = 293 K
β = 94.704 (2)°	Block, colorless
*V* = 3530.33 (15) Å^3^	0.3 × 0.2 × 0.2 mm
*Z* = 8	

### Data collection

**Table:**

Oxford Diffraction Xcalibur Sapphire3 diffractometer	3467 independent reflections
Radiation source: fine-focus sealed tube	2538 reflections with *I* > 2σ(*I*)
Graphite monochromator	*R*_int_ = 0.065
Detector resolution: 16.1049 pixels mm^-1^	θ_max_ = 26.0°, θ_min_ = 3.4°
ω scans	*h* = −29→29
Absorption correction: multi-scan (*CrysAlis PRO*; Oxford Diffraction, 2010)	*k* = −11→11
*T*_min_ = 0.766, *T*_max_ = 1.000	*l* = −19→19
40937 measured reflections	

### Refinement

**Table:**

Refinement on *F*^2^	Primary atom site location: structure-invariant direct methods
Least-squares matrix: full	Secondary atom site location: difference Fourier map
*R*[*F*^2^ > 2σ(*F*^2^)] = 0.061	Hydrogen site location: inferred from neighbouring sites
*wR*(*F*^2^) = 0.126	H-atom parameters constrained
*S* = 1.03	*w* = 1/[σ^2^(*F*_o_^2^) + (0.0389*P*)^2^ + 5.1587*P*] where *P* = (*F*_o_^2^ + 2*F*_c_^2^)/3
3467 reflections	(Δ/σ)_max_ = 0.001
258 parameters	Δρ_max_ = 0.26 e Å^−^^3^
10 restraints	Δρ_min_ = −0.32 e Å^−^^3^

### Special details

**Table:**

Experimental. CrysAlisPro, Oxford Diffraction Ltd., Version 1.171.34.40 (release 27-08-2010 CrysAlis171 .NET) (compiled Aug 27 2010,11:50:40) Empirical absorption correction using spherical harmonics, implemented in SCALE3 ABSPACK scaling algorithm.
Geometry. All esds (except the esd in the dihedral angle between two l.s. planes) are estimated using the full covariance matrix. The cell esds are taken into account individually in the estimation of esds in distances, angles and torsion angles; correlations between esds in cell parameters are only used when they are defined by crystal symmetry. An approximate (isotropic) treatment of cell esds is used for estimating esds involving l.s. planes.
Refinement. Refinement of F^2^ against ALL reflections. The weighted R-factor wR and goodness of fit S are based on F^2^, conventional R-factors R are based on F, with F set to zero for negative F^2^. The threshold expression of F^2^ > 2sigma(F^2^) is used only for calculating R-factors(gt) etc. and is not relevant to the choice of reflections for refinement. R-factors based on F^2^ are statistically about twice as large as those based on F, and R- factors based on ALL data will be even larger.

### Fractional atomic coordinates and isotropic or equivalent isotropic displacement parameters (Å^2^)

**Table:**

	*x*	*y*	*z*	*U*_iso_*/*U*_eq_	Occ. (<1)
O1	0.08902 (7)	0.55303 (15)	1.02901 (8)	0.0401 (4)	
C2	0.06233 (9)	0.6806 (2)	1.01297 (12)	0.0324 (5)	
O2	0.06995 (8)	0.41341 (18)	0.74350 (9)	0.0506 (5)	
C3	0.04766 (9)	0.7238 (2)	0.93258 (12)	0.0308 (5)	
C4	0.06583 (9)	0.6445 (2)	0.85626 (12)	0.0319 (5)	
H4	0.0335	0.6405	0.8138	0.038*	
C4A	0.08096 (9)	0.4945 (2)	0.88253 (12)	0.0313 (5)	
C5	0.08182 (9)	0.3842 (2)	0.81766 (13)	0.0358 (5)	
C6	0.09581 (11)	0.2348 (2)	0.84666 (15)	0.0431 (6)	
H6A	0.1117	0.1836	0.8011	0.052*	
H6B	0.0612	0.1869	0.8588	0.052*	
C7	0.13766 (10)	0.2287 (2)	0.92539 (14)	0.0394 (5)	
C8	0.11327 (11)	0.3177 (2)	0.99442 (14)	0.0402 (6)	
H8A	0.0821	0.2663	1.0161	0.048*	
H8B	0.1421	0.3309	1.0407	0.048*	
C8A	0.09300 (9)	0.4593 (2)	0.96338 (13)	0.0319 (5)	
C9	0.11372 (10)	0.7223 (2)	0.81771 (12)	0.0337 (5)	
C10	0.16874 (10)	0.7180 (2)	0.85477 (13)	0.0377 (5)	
H10	0.1772	0.6617	0.9023	0.045*	
C11	0.21092 (11)	0.7965 (3)	0.82186 (15)	0.0454 (6)	
C12	0.19901 (13)	0.8810 (3)	0.75148 (17)	0.0575 (7)	
H12	0.2275	0.9335	0.7292	0.069*	
C13	0.14458 (14)	0.8865 (3)	0.71472 (16)	0.0636 (8)	
H13	0.1360	0.9440	0.6677	0.076*	
C14	0.10264 (11)	0.8071 (3)	0.74738 (14)	0.0498 (7)	
H14	0.0661	0.8107	0.7214	0.060*	
C15	0.26941 (13)	0.7941 (4)	0.8631 (2)	0.0643 (8)	
C19	0.01734 (9)	0.8518 (2)	0.91848 (12)	0.0328 (5)	
N20	−0.00772 (9)	0.9545 (2)	0.90387 (12)	0.0495 (6)	
N21	0.05451 (9)	0.7470 (2)	1.08480 (11)	0.0446 (5)	
H21A	0.0381	0.8288	1.0839	0.054*	
H21B	0.0658	0.7083	1.1323	0.054*	
C22	0.19433 (11)	0.2886 (3)	0.90370 (18)	0.0583 (7)	
H22A	0.1887	0.3805	0.8779	0.088*	
H22B	0.2109	0.2254	0.8652	0.088*	
H22C	0.2190	0.2975	0.9544	0.088*	
C23	0.14521 (13)	0.0752 (3)	0.95659 (17)	0.0592 (8)	
H23A	0.1580	0.0171	0.9122	0.089*	
H23B	0.1098	0.0395	0.9727	0.089*	
H23C	0.1726	0.0727	1.0044	0.089*	
F1A	0.2811 (5)	0.8968 (12)	0.9208 (6)	0.089 (2)	0.507 (7)
F2A	0.2806 (3)	0.6766 (7)	0.9151 (5)	0.0813 (17)	0.507 (7)
F3A	0.3113 (2)	0.7874 (11)	0.8116 (4)	0.091 (2)	0.507 (7)
F1B	0.2694 (8)	0.861 (2)	0.9364 (9)	0.089 (2)	0.330 (7)
F2B	0.2877 (5)	0.6587 (9)	0.8792 (8)	0.0813 (17)	0.330 (7)
F3B	0.3049 (4)	0.8668 (14)	0.8182 (7)	0.091 (2)	0.330 (7)
F1C	0.2904 (6)	0.9254 (11)	0.8551 (10)	0.089 (2)	0.163 (3)
F2C	0.2730 (6)	0.7466 (19)	0.9427 (6)	0.0813 (17)	0.163 (3)
F3C	0.2982 (6)	0.7011 (16)	0.8215 (10)	0.091 (2)	0.163 (3)

### Atomic displacement parameters (Å^2^)

**Table:**

	*U*^11^	*U*^22^	*U*^33^	*U*^12^	*U*^13^	*U*^23^
O1	0.0604 (11)	0.0333 (8)	0.0262 (7)	0.0141 (7)	0.0006 (7)	−0.0023 (6)
C2	0.0373 (13)	0.0274 (10)	0.0326 (11)	0.0049 (9)	0.0042 (9)	−0.0011 (9)
O2	0.0679 (12)	0.0506 (10)	0.0325 (9)	0.0070 (9)	−0.0012 (8)	−0.0119 (8)
C3	0.0333 (12)	0.0291 (10)	0.0299 (11)	0.0049 (9)	0.0027 (8)	−0.0011 (8)
C4	0.0365 (13)	0.0338 (11)	0.0246 (10)	0.0056 (9)	−0.0024 (8)	−0.0015 (8)
C4A	0.0303 (12)	0.0325 (11)	0.0311 (11)	0.0007 (9)	0.0023 (8)	−0.0036 (9)
C5	0.0316 (12)	0.0403 (12)	0.0358 (12)	−0.0004 (10)	0.0042 (9)	−0.0079 (10)
C6	0.0495 (15)	0.0323 (12)	0.0480 (13)	−0.0001 (11)	0.0073 (11)	−0.0114 (10)
C7	0.0436 (14)	0.0307 (11)	0.0450 (13)	0.0061 (10)	0.0101 (10)	−0.0006 (10)
C8	0.0524 (15)	0.0311 (11)	0.0377 (12)	0.0061 (11)	0.0075 (10)	0.0026 (10)
C8A	0.0344 (13)	0.0288 (11)	0.0328 (11)	0.0009 (9)	0.0055 (9)	−0.0025 (9)
C9	0.0438 (14)	0.0323 (11)	0.0255 (10)	0.0066 (10)	0.0057 (9)	−0.0034 (9)
C10	0.0438 (14)	0.0376 (12)	0.0322 (11)	0.0059 (10)	0.0058 (10)	0.0014 (10)
C11	0.0484 (15)	0.0464 (14)	0.0431 (13)	0.0029 (12)	0.0151 (11)	−0.0079 (11)
C12	0.063 (2)	0.0597 (17)	0.0531 (16)	−0.0032 (14)	0.0267 (14)	0.0068 (13)
C13	0.078 (2)	0.0712 (19)	0.0429 (15)	0.0045 (16)	0.0142 (14)	0.0259 (14)
C14	0.0539 (17)	0.0607 (16)	0.0345 (13)	0.0068 (13)	0.0022 (11)	0.0116 (12)
C15	0.0492 (18)	0.077 (2)	0.0679 (19)	−0.0027 (16)	0.0137 (14)	0.0023 (17)
C19	0.0381 (13)	0.0354 (12)	0.0248 (10)	0.0012 (10)	0.0023 (9)	−0.0033 (9)
N20	0.0636 (15)	0.0412 (12)	0.0423 (11)	0.0166 (11)	−0.0040 (10)	−0.0042 (9)
N21	0.0708 (15)	0.0362 (10)	0.0270 (9)	0.0175 (10)	0.0058 (9)	−0.0005 (8)
C22	0.0380 (15)	0.0703 (18)	0.0675 (17)	0.0098 (14)	0.0094 (12)	0.0119 (15)
C23	0.080 (2)	0.0344 (13)	0.0647 (17)	0.0141 (13)	0.0142 (15)	−0.0008 (12)
F1A	0.061 (5)	0.119 (6)	0.085 (3)	−0.017 (3)	−0.006 (3)	−0.031 (5)
F2A	0.057 (3)	0.098 (3)	0.089 (5)	0.029 (2)	0.005 (3)	−0.004 (3)
F3A	0.0386 (17)	0.154 (7)	0.0855 (18)	0.008 (3)	0.0329 (12)	0.012 (4)
F1B	0.061 (5)	0.119 (6)	0.085 (3)	−0.017 (3)	−0.006 (3)	−0.031 (5)
F2B	0.057 (3)	0.098 (3)	0.089 (5)	0.029 (2)	0.005 (3)	−0.004 (3)
F3B	0.0386 (17)	0.154 (7)	0.0855 (18)	0.008 (3)	0.0329 (12)	0.012 (4)
F1C	0.061 (5)	0.119 (6)	0.085 (3)	−0.017 (3)	−0.006 (3)	−0.031 (5)
F2C	0.057 (3)	0.098 (3)	0.089 (5)	0.029 (2)	0.005 (3)	−0.004 (3)
F3C	0.0386 (17)	0.154 (7)	0.0855 (18)	0.008 (3)	0.0329 (12)	0.012 (4)

### Geometric parameters (Å, º)

**Table:**

O1—C2	1.369 (2)	C11—C12	1.381 (4)
O1—C8A	1.373 (2)	C11—C15	1.487 (4)
C2—N21	1.327 (3)	C12—C13	1.375 (4)
C2—C3	1.358 (3)	C12—H12	0.9300
O2—C5	1.220 (3)	C13—C14	1.379 (4)
C3—C19	1.409 (3)	C13—H13	0.9300
C3—C4	1.515 (3)	C14—H14	0.9300
C4—C4A	1.504 (3)	C15—F3C	1.320 (8)
C4—C9	1.522 (3)	C15—F1B	1.321 (8)
C4—H4	0.9800	C15—F3B	1.336 (7)
C4A—C8A	1.334 (3)	C15—F2C	1.337 (8)
C4A—C5	1.462 (3)	C15—F3A	1.339 (5)
C5—C6	1.505 (3)	C15—F1C	1.340 (8)
C6—C7	1.534 (3)	C15—F1A	1.344 (5)
C6—H6A	0.9700	C15—F2B	1.360 (7)
C6—H6B	0.9700	C15—F2A	1.389 (5)
C7—C22	1.524 (3)	C19—N20	1.147 (3)
C7—C23	1.529 (3)	N21—H21A	0.8600
C7—C8	1.530 (3)	N21—H21B	0.8600
C8—C8A	1.484 (3)	C22—H22A	0.9600
C8—H8A	0.9700	C22—H22B	0.9600
C8—H8B	0.9700	C22—H22C	0.9600
C9—C14	1.379 (3)	C23—H23A	0.9600
C9—C10	1.389 (3)	C23—H23B	0.9600
C10—C11	1.380 (3)	C23—H23C	0.9600
C10—H10	0.9300		
			
C2—O1—C8A	118.60 (15)	C9—C14—C13	121.4 (2)
N21—C2—C3	128.71 (19)	C9—C14—H14	119.3
N21—C2—O1	110.26 (17)	C13—C14—H14	119.3
C3—C2—O1	121.03 (18)	F3C—C15—F1B	142.4 (12)
C2—C3—C19	119.51 (18)	F3C—C15—F3B	72.2 (8)
C2—C3—C4	122.53 (18)	F1B—C15—F3B	105.9 (9)
C19—C3—C4	117.84 (17)	F3C—C15—F2C	104.9 (10)
C4A—C4—C3	108.37 (16)	F3B—C15—F2C	132.9 (9)
C4A—C4—C9	113.05 (18)	F1B—C15—F3A	127.9 (8)
C3—C4—C9	110.95 (17)	F2C—C15—F3A	124.6 (7)
C4A—C4—H4	108.1	F3C—C15—F1C	110.4 (10)
C3—C4—H4	108.1	F1B—C15—F1C	71.1 (10)
C9—C4—H4	108.1	F2C—C15—F1C	113.6 (10)
C8A—C4A—C5	119.27 (19)	F3A—C15—F1C	71.6 (8)
C8A—C4A—C4	121.77 (18)	F3C—C15—F1A	136.9 (9)
C5—C4A—C4	118.96 (17)	F3B—C15—F1A	83.9 (6)
O2—C5—C4A	120.4 (2)	F2C—C15—F1A	66.4 (10)
O2—C5—C6	122.18 (19)	F3A—C15—F1A	109.1 (5)
C4A—C5—C6	117.37 (18)	F1B—C15—F2B	107.4 (12)
C5—C6—C7	113.39 (18)	F3B—C15—F2B	111.8 (7)
C5—C6—H6A	108.9	F2C—C15—F2B	61.1 (8)
C7—C6—H6A	108.9	F3A—C15—F2B	80.2 (5)
C5—C6—H6B	108.9	F1C—C15—F2B	139.6 (8)
C7—C6—H6B	108.9	F1A—C15—F2B	119.8 (9)
H6A—C6—H6B	107.7	F3C—C15—F2A	72.0 (8)
C22—C7—C23	109.8 (2)	F1B—C15—F2A	82.3 (11)
C22—C7—C8	110.7 (2)	F3B—C15—F2A	128.6 (6)
C23—C7—C8	108.87 (19)	F3A—C15—F2A	102.1 (4)
C22—C7—C6	109.1 (2)	F1C—C15—F2A	137.0 (7)
C23—C7—C6	110.5 (2)	F1A—C15—F2A	98.3 (7)
C8—C7—C6	107.79 (19)	F3C—C15—C11	107.1 (7)
C8A—C8—C7	112.50 (18)	F1B—C15—C11	108.2 (9)
C8A—C8—H8A	109.1	F3B—C15—C11	111.4 (6)
C7—C8—H8A	109.1	F2C—C15—C11	114.0 (7)
C8A—C8—H8B	109.1	F3A—C15—C11	116.4 (4)
C7—C8—H8B	109.1	F1C—C15—C11	106.6 (6)
H8A—C8—H8B	107.8	F1A—C15—C11	115.0 (6)
C4A—C8A—O1	123.33 (18)	F2B—C15—C11	111.7 (6)
C4A—C8A—C8	125.43 (19)	F2A—C15—C11	113.6 (4)
O1—C8A—C8	111.23 (17)	N20—C19—C3	177.4 (2)
C14—C9—C10	118.1 (2)	C2—N21—H21A	120.0
C14—C9—C4	120.2 (2)	C2—N21—H21B	120.0
C10—C9—C4	121.58 (18)	H21A—N21—H21B	120.0
C11—C10—C9	120.7 (2)	C7—C22—H22A	109.5
C11—C10—H10	119.6	C7—C22—H22B	109.5
C9—C10—H10	119.6	H22A—C22—H22B	109.5
C10—C11—C12	120.4 (2)	C7—C22—H22C	109.5
C10—C11—C15	120.4 (2)	H22A—C22—H22C	109.5
C12—C11—C15	119.2 (2)	H22B—C22—H22C	109.5
C13—C12—C11	119.2 (2)	C7—C23—H23A	109.5
C13—C12—H12	120.4	C7—C23—H23B	109.5
C11—C12—H12	120.4	H23A—C23—H23B	109.5
C12—C13—C14	120.2 (2)	C7—C23—H23C	109.5
C12—C13—H13	119.9	H23A—C23—H23C	109.5
C14—C13—H13	119.9	H23B—C23—H23C	109.5
			
C8A—O1—C2—N21	170.12 (19)	C7—C8—C8A—O1	−159.98 (19)
C8A—O1—C2—C3	−9.9 (3)	C4A—C4—C9—C14	138.3 (2)
N21—C2—C3—C19	−3.7 (4)	C3—C4—C9—C14	−99.8 (2)
O1—C2—C3—C19	176.4 (2)	C4A—C4—C9—C10	−46.0 (3)
N21—C2—C3—C4	172.3 (2)	C3—C4—C9—C10	76.0 (2)
O1—C2—C3—C4	−7.7 (3)	C14—C9—C10—C11	0.0 (3)
C2—C3—C4—C4A	21.0 (3)	C4—C9—C10—C11	−175.90 (19)
C19—C3—C4—C4A	−163.03 (19)	C9—C10—C11—C12	0.2 (3)
C2—C3—C4—C9	−103.7 (2)	C9—C10—C11—C15	178.5 (2)
C19—C3—C4—C9	72.3 (2)	C10—C11—C12—C13	0.3 (4)
C3—C4—C4A—C8A	−19.2 (3)	C15—C11—C12—C13	−178.1 (3)
C9—C4—C4A—C8A	104.2 (2)	C11—C12—C13—C14	−0.8 (4)
C3—C4—C4A—C5	160.06 (18)	C10—C9—C14—C13	−0.5 (4)
C9—C4—C4A—C5	−76.5 (2)	C4—C9—C14—C13	175.4 (2)
C8A—C4A—C5—O2	178.7 (2)	C12—C13—C14—C9	0.9 (4)
C4—C4A—C5—O2	−0.7 (3)	C10—C11—C15—F3C	97.7 (9)
C8A—C4A—C5—C6	0.7 (3)	C12—C11—C15—F3C	−83.9 (9)
C4—C4A—C5—C6	−178.6 (2)	C10—C11—C15—F1B	−69.1 (11)
O2—C5—C6—C7	149.3 (2)	C12—C11—C15—F1B	109.3 (10)
C4A—C5—C6—C7	−32.8 (3)	C10—C11—C15—F3B	174.8 (7)
C5—C6—C7—C22	−65.0 (3)	C12—C11—C15—F3B	−6.8 (8)
C5—C6—C7—C23	174.1 (2)	C10—C11—C15—F2C	−17.9 (10)
C5—C6—C7—C8	55.3 (3)	C12—C11—C15—F2C	160.5 (9)
C22—C7—C8—C8A	71.6 (3)	C10—C11—C15—F3A	138.6 (6)
C23—C7—C8—C8A	−167.6 (2)	C12—C11—C15—F3A	−43.0 (6)
C6—C7—C8—C8A	−47.7 (3)	C10—C11—C15—F1C	−144.1 (8)
C5—C4A—C8A—O1	−174.82 (19)	C12—C11—C15—F1C	34.3 (9)
C4—C4A—C8A—O1	4.5 (3)	C10—C11—C15—F1A	−91.9 (6)
C5—C4A—C8A—C8	6.5 (3)	C12—C11—C15—F1A	86.5 (6)
C4—C4A—C8A—C8	−174.2 (2)	C10—C11—C15—F2B	49.0 (7)
C2—O1—C8A—C4A	11.7 (3)	C12—C11—C15—F2B	−132.6 (6)
C2—O1—C8A—C8	−169.41 (19)	C10—C11—C15—F2A	20.3 (5)
C7—C8—C8A—C4A	18.8 (3)	C12—C11—C15—F2A	−161.3 (4)

### Hydrogen-bond geometry (Å, º)

**Table:**

*D*—H···*A*	*D*—H	H···*A*	*D*···*A*	*D*—H···*A*
N21—H21*A*···N20^i^	0.86	2.17	3.025 (3)	171
N21—H21*B*···O2^ii^	0.86	2.10	2.934 (2)	163

Symmetry codes: (i) −*x*, −*y*+2, −*z*+2; (ii) *x*, −*y*+1, *z*+1/2.
